# A novel pulse-current waveform circuit for low-energy consumption and low-noise transcranial magnetic stimulation

**DOI:** 10.3389/fnins.2024.1500619

**Published:** 2025-01-07

**Authors:** Xinhua Tan, Ao Guo, Jiasheng Tian, Yingwei Li, Jian Shi

**Affiliations:** ^1^School of Information Science and Engineering, Yanshan University, Qinhuangdao, China; ^2^School of Electronic Information and Communications, Huazhong University of Science and Technology, Wuhan City, China; ^3^Hebei Key Laboratory of Information Transmission and Signal Processing, Qinhuangdao, China

**Keywords:** transcranial magnetic stimulation, low energy consumption, low noise, asymmetrical pulse waveform, pulse generation circuit

## Abstract

**Introduction:**

Transcranial magnetic stimulation (TMS) is widely used for the noninvasive activation of neurons in the human brain. It utilizes a pulsed magnetic field to induce electric pulses that act on the central nervous system, altering the membrane potential of nerve cells in the cerebral cortex to treat certain mental diseases. However, the effectiveness of TMS can be compromised by significant heat generation and the clicking noise produced by the pulse in the TMS coil. This study proposes a novel, non-resonant, high-frequency switching design controlled by high-frequency pulse-width modulation (PWM) voltage excitation to achieve ideal pulse-current waveforms that minimize both clicking noise and heat generation from the TMS coil.

**Method:**

First, a particle swarm optimization algorithm was used to optimize the pulse-current waveform, minimizing both the resistance loss and clicking noise (vibration energy) generated by the TMS coils. Next, the pulse-current waveform was modeled based on the principles of programmable transcranial magnetic stimulation circuits. The relationships between the parameters of the pulse-current waveform, vibration energy, and ohmic resistance loss in the TMS coil were explored, ensuring the necessary depolarization of the nerve membrane potential. Finally, four insulated-gate bipolar transistors, controlled by a series of PWM pulse sequences, generated the desired pulse-current duration and direction in the H-bridge circuit. The duration and slope of the rising and falling segments of the current waveform were adjusted by the PWM pulse duration.

**Results:**

The optimized current waveform, represented by three segmented functions, reduces heat loss and noise while inducing a greater change in neural membrane potential compared with those obtained with conventional symmetric waveforms. Spectral analysis further confirmed that the noise spectrum of the optimized current waveform, particularly the peak spectrum, is significantly lower than that of the conventional triangular symmetric waveform.

**Conclusion:**

The study provide a method and new ideas for low energy consumption and low-noise transcranial magnetic stimulation by using TMS circuit design techniques as well as waveform optimization.

## 1 Introduction

Transcranial magnetic stimulation (TMS) is a noninvasive technology used to stimulate and modulate neurons in the brain. It is widely applied in the treatment of major depressive disorder, Parkinson’s disease, post-traumatic stress disorder, and other neurological conditions such as acute ischemic stroke (Harel et al., 2014; Spagnolo et al., 2021; Petrosino et al., 2021; Peruzzotti-Jametti et al., 2013; Boniface, 2001). Merton and Morton (1980) proposed a high-voltage stimulator, known as transcranial electrical stimulation, which activates the cerebral cortex. Since then, transcranial direct current stimulation has been used in clinical applications. However, transcranial direct current stimulation often causes discomfort and tingling at the electrode site. Baker (1985) proposed a painless, noninvasive method that uses alternating electromagnetic fields to stimulate brain tissue and alter the excitability of neurons; this method, which has since been approved by the International Joint Conference in Clinical Neurophysiology, is now known as TMS. The pulsed current in a TMS coil induces an electric field in the human brain, which acts on the neuronal membrane and depolarizes it. When the induced electric field reaches the activation threshold, neurons discharge and are fully stimulated (Jin et al., 2012). Over the past 30 years, TMS has become a valuable tool in clinical physiology and is widely used in neuroscience, particularly as an experimental intervention for depression and other psychiatric and neurological disorders (Hallett, 2007; Perera et al., 2016; Wassermann et al., 2008; Crowther, 2014).

However, TMS devices, particularly the TMS coil, which runs at short, high-current pulses (thousands of amperes), generate significant heat and vibration (Hsu et al., 2003). These factors lead to energy loss and loud clicking noises, which are closely related to the pulse-current waveforms generated in TMS circuits (Tringali et al., 2012). Presently available TMS devices, particularly pulse circuit generators, offer limited pulse-current waveform options. Consequently, the heat and noise generated by TMS coils remain a challenge, reducing the effectiveness of TMS treatments for nervous system diseases (Zhang et al., 2023).

Historically, monophasic and biphasic pulse currents were first proposed to effectively depolarize neurons in the cerebral cortex and generate action-and motor-evoked potentials in the motor areas of the cortex. While monophasic pulses have advantages in neural activation (Arai et al., 2007; Goetz et al., 2016; Kammer et al., 2001; Niehaus et al., 2000; Sommer et al., 2006; Taylor and Loo, 2007), biphasic waveforms are more commonly used in clinical practice as they are more effective for treating mental disorders (Goetz et al., 2012a, 2012b, 2013; Niehaus et al., 2000). However, to improve TMS effectiveness and flexibility, in addition to redesigning the TMS coil structure and its materials, and to decrease the TMS coil ohmic loss and clicking noise, the biphasic pulse-current waveform will have to be optimized.

In the past decade, many researchers (Gattinger et al., 2012; Peterchev et al., 2014; Sorkhabi et al., 2021a, 2022; Li et al., 2022) have devoted themselves to optimizing the circuit topology structure to achieve multiple pulse waveforms for improved stimulation effectiveness, flexibility, etc. Gattinger et al. (2012) proposed a novel repetitive transcranial magnetic stimulation device called FlexTMS, which used a full bridge circuit structure, four insulated gate bipolar transistor (IGBT) modules, and an energy storage capacitor. The FlexTMS system could achieve a wider range of controllable parameters, including pulse width, polarity, intensity, and variable interval dual pulse sequences. However, the device was inapplicable for high-repetition pulses and was sensitive to changes in the coil inductance. Peterchev et al. (2014) presented a third-generation controllable pulse parameter device that used a novel circuit topology with two energy-storage capacitors and accomplished more flexible pulse shaping; however, the device also exhibited long decay of the coil current, resulting in greater heat dissipation in the TMS coil and in the IGBT modules. Sorkhabi et al. (2021a) proposed a second-generation programmable TMS device that used cascaded H-bridge inverters and phase-shifted pulse-width modulation (PWM); it could generate highly adjustable magnetic pulses in terms of waveforms, polarities, and patterns. Their paper (Sorkhabi et al., 2021a) demonstrated that increasing the number of PWM voltage levels from 3 to 5 could have a significant positive effect on the PWM-based TMS pulse and membrane voltage changes; however, increasing further from a 5-level PWM system up to a 7-level PWM system did not yield any notable improvement. Moreover, the device with the 7-level PWM system was complex and costly to build. Nonetheless, despite its limitations, it presented an idea for obtaining arbitrary and highly customizable magnetic pulses. Sorkhabi et al. (2022) achieved a wider range of pulse waveforms by using PWM (known as programmable TMS or pTMS). Their paper (Sorkhabi et al., 2022) demonstrated how PWM provided flexible waveform control for achieving diverse required pulse outputs. However, their proposed device was limited to lower stimulation amplitudes.

Li et al. (2022) presented a modular pulse synthesizer that could, for the first time, flexibly generate high-power TMS pulses with user-defined electric field shapes and rapid pulse sequences with high output quality.

To reduce energy consumption and heat loss in TMS devices, Peterchev et al. (2007) proposed a device with controllable pulse width (PW) that could generate near-rectangular induced electric field pulses while using 2–34% less energy and 67–72% less coil heating compared to those required for matched conventional cosine pulses. In their study (Peterchev et al., 2007), the coil heating was estimated based on the load integral of the coil current; however, the effects of the pulse shape were not taken into account. Wang et al. (2023) proposed an improved single-phase pulse waveform that significantly reduced coil heating while maintaining the original electric field characteristics, thus making single-phase TMS more feasible at high frequencies.

To reduce TMS noise, Goetz et al. (2014) redesigned both the pulse waveform and the coil structure. In their study (Goetz et al., 2014), they used ultra-brief current pulses (down to 45-μs biphasic duration) to drive a prototype coil and reduced the peak sound pressure level by more than 25 dB compared to those obtained using a conventional TMS configuration. Their proposed higher-voltage devices could also shift a higher portion of the sound spectrum above the human hearing range. Additionally, they introduced an improved mechanical structure for the TMS coil to suppress sound at the source, diminish down-mixing of high-frequency sound into the audible range, and impede the transmission of residual sound to the coil surface, among others (Goetz et al., 2014). However, Goetz et al. (2014) did not consider the effects of the shape of the current pulse. Because of electromagnetic forces, the coil produces clicking sounds, which may cause hearing damage (Dhamne et al., 2014; Tringali et al., 2012). At the same time, because of the nonlinearity and the large number of interactions in the brain, the click deteriorates the focus of the TMS (Goetz et al., 2014; Siebner et al., 1999). The noise may also cause the patient to become restless, upset, and anxious, which could greatly and adversely impact the treatment effect (Dhamne et al., 2014).

By contrast, conventional pulse-current waveforms, such as traditional sine or symmetric pulse waveforms, have been studied and modeled, and optimization algorithms, such as particle swarm optimization and genetic algorithms, have been used to obtain optimized waveforms under limited conditions (Zhang et al., 2023). For example, particle swarm optimization has been used to optimize a traditional sine pulse wave (Zhang et al., 2023). Numerous optimized waveforms have been proposed from both theoretical and electromagnetic simulation perspectives. However, it is difficult to satisfy pulse-current circuit structure and design requirements using the proposed pulse-current waveforms. To illustrate, in the aforementioned study (Zhang et al., 2023), the pulse circuit of the optimized asymmetric waveform was designed, and its corresponding parameters had to be defined and set in detail. In addition, the current TMS drive system, which uses a resistor–inductor circuit for charging and discharging to generate a pulsed current, lacks flexibility and tunability.

Therefore, this study introduces a controlled high-frequency switching structure that uses four IGBTs, significantly enhancing the flexibility and tunability of pulse-current waveforms. The established circuit model in this study is somewhat similar to that provided by Sorkhabi et al. (2021b), but the editable compilers and the methods of controlling IGBT are greatly different from those provided by Sorkhabi et al. (2021b) and other scholars (Gattinger et al., 2012; Peterchev et al., 2014; Sorkhabi et al., 2021a; Sorkhabi et al., 2022). Firstly, according to the objectives of the low-energy consumption and low-noise TMS, the parameters of the requested (or fitted) current waveform in the TMS coil were found out by applying the particle swarm algorithm. Secondly, the four PWM compilers are programmed in terms of the requested current waveform and their parameters including the parameters of the PWM waveforms (duty cycle, period and duration). Finally, the four PWM compilers generate a series of PWM pulse sequences to operate the switches of IGBTs for obtaining the requested current waveform. However, Sorkhabi et al. (2021b) obtained switching states for each switch in the H-bridge by comparing the reference signal (V_rf_) with the carrier signal (V_c_). Due to the operating frequency of the triangular waveform and complex relationships between the carrier signal (or the triangular waveform) and the reference signal (to be simulated by the PWM), the adjustable width of PWM was limited so that the generated current waveform is approximately close to the requested stimulus waveform in a certain degree. In this study, the obtained current waveform is more closer to the requested current waveform than that provided by Sorkhabi et al. (2021b). The novel methods are the programmable PWM compilers and the relationships between the requested (or fitted) waveform and the PWM waveforms, including their respective parameters. This circuit enables the generation of various pulse waveforms, providing new opportunities to reduce TMS ohmic losses and clicking noises.

## 2 Materials and methods

### 2.1 Circuit model

The programmable transcranial magnetic stimulation (pTMS) system combines a non-resonant, high-frequency switching architecture with the low-pass filtering properties of nerve cells to synthesize the required waveforms. This modulation technique allows precise control over the pulse-current waveform, frequency, mode, and stimulus intensity (Sorkhabi et al., 2021b). The pTMS system consists of three stages, during which electric power is transformed into a high-power magnetic pulse to generate an induced electric field in the human brain. The first stage is the step-up of the transformer. This stage increases the voltage to the desired level using a transformer. In the second stage, the full-wave rectifier bridge system converts the alternating current (AC) voltage into a direct current (DC) voltage, including an electric field energy storage bank in the form of a DC container. In the third stage, pulse-width modulation (PWM) excitation signals are generated from four programmable editors to control the on and off states of the four insulated gate bipolar transistors (IGBTs). The PWM voltage signals control the four IGBTs on the H-bridge such that they work together to control the voltage and current on the TMS coil, resulting in a time-varying magnetic field generated by the current-carrying TMS coil.

**Figure 1:**
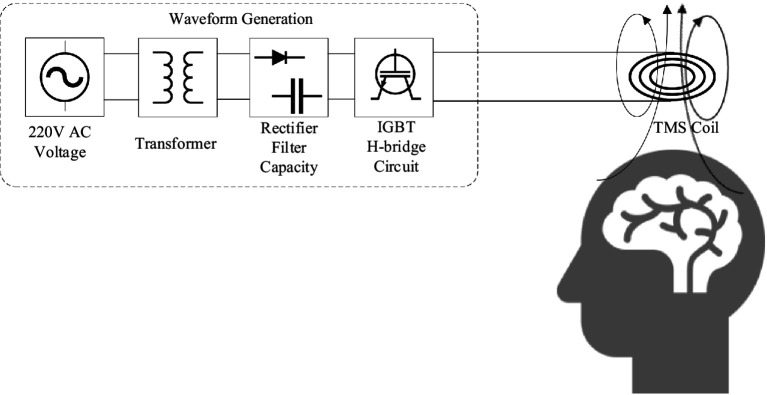
Conceptual diagram of a pulse drive system for pTMS.

**Figure 2:**
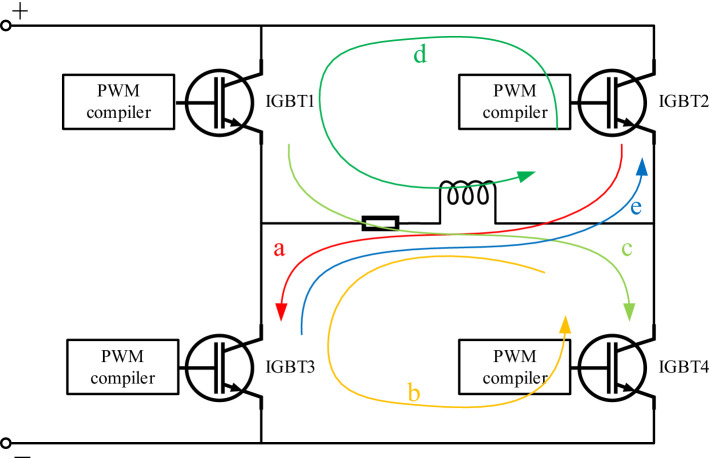
Programmable H-bridge circuit.

A concept diagram of the pTMS pulse drive system, consisting of an AC voltage source, a transformer, a rectifier, an energy storage module, and an H-bridge circuit, is shown in Figure 1. In Figure 1, the AC voltage is transformed to the required voltage by the transformer, and then a full-wave rectifier bridge converts the AC voltage to DC voltage, where the electric energy is stored in a DC capacitor. Finally, a PWM voltage is excited from the DC capacitor by operating the switches of IGBTs in terms of a series of PWM pulse sequences controlled by the PWM compilers. The key component of the circuit system is the H-bridge circuit, which consists of four IGBTs controlled by four programming devices (PWM compiler, illustrated in Figure 2). In Figure 2, when IGBT2 and IGBT3 are turned on, IGBT1 and IGBT4 are turned off, the TMS coil current is shown as streamline a. When only IGBT3 is turned on, the current is shown as streamline b. When only IGBT1 and IGBT4 are turned on, the coil current is shown as streamline c. When only IGBT1 is turned on, the coil current is shown as streamline d. In order to make the coil current become zero, all IGBTs are turned off, resulting in the coil current shown by streamline e. In this study, the TMS coil resistance was set to 0.05 *Ω* and its inductance to 20 μH (Peterchev et al., 2014; Zeng et al., 2022), which could match the standard TMS coil in a great degree.

**Figure 3:**
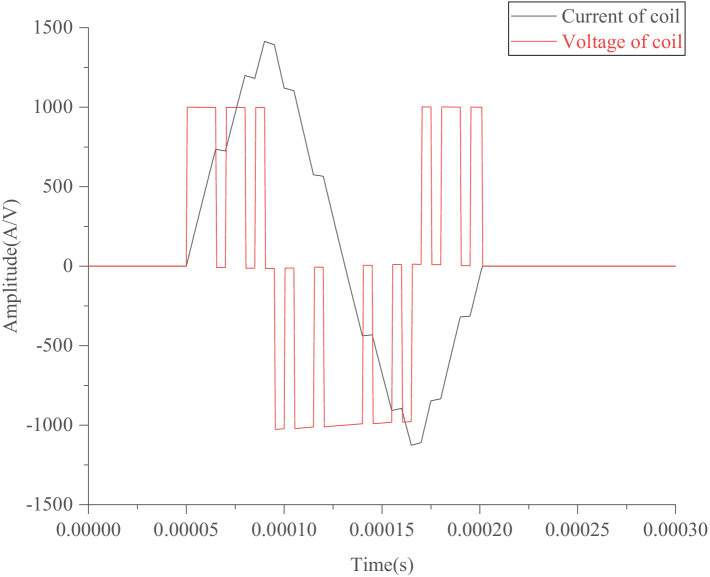
Simulated sine wave.

**Figure 4:**
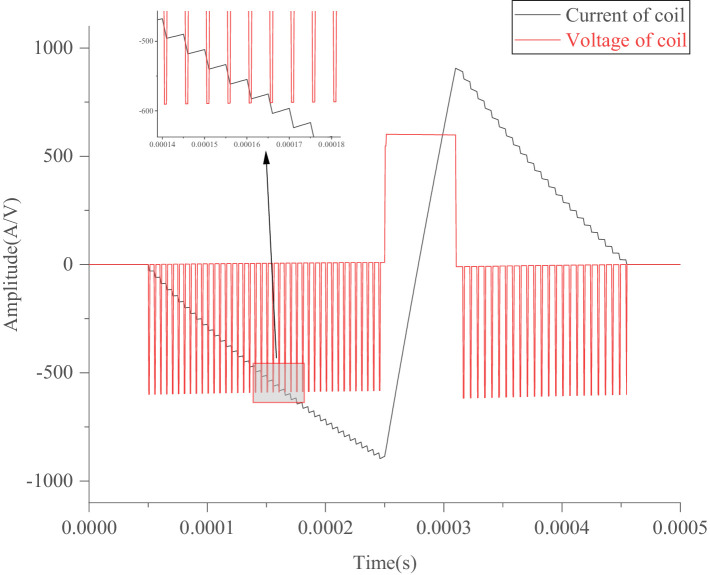
Simulated symmetrical waveforms.

Through modifications on a series of codes in the programming module connected to the IGBTs, four specific sequences of PWM voltage waveforms were generated to control the four IGBT switches. This configuration enables the generation of the required pulse-current waveform on the TMS coil, as illustrated in Figures 3, 4.

Figure 3 shows an approximate sine wave simulated using a pTMS circuit system based on the established model in MATLAB Simulink. In this study, as shown in Figure 2, the Simulink module in MATLAB (R2012b) was used to simulate IGBT circuits, in which a module for PWM generates pulse sequences to control the on and off states of the IGBTs using editable MATLAB functions. As shown in Figure 3, the sine wave consists of 11 impulse voltage excitations with widths ranging from 5 μs to 15 μs, producing a pulse-current waveform that resembles a sinusoidal pulse waveform. Figure 4 illustrates the optimized waveform obtained using particle swarm optimization, as described in the paper by Zhang et al. (2023). In this study, the particle swarm algorithm is used to obtain the parameters of the PWM waveform (duty cycle, period and duration) according to the set objectives, and then in terms of these parameters the programmable PWM compilers are edited to generate a series of PWM pulse sequences to operate the switches of IGBTs for obtaining the requested current waveform. The waveform shown in Figure 4 is a symmetrical triangular waveform. From the established circuit in this study, the optimized waveform was excited by 40 + 36 narrow pulses with a 5 μs width and one wide pulse with a 60 μs width, with duty ratios of 0.2 and 0.167, respectively. These narrow and wide pulses and their combinations can flexibly generate various pulse-current waveforms on the TMS coil to fulfil expected requirements, such as reduced noise and ohmic loss.

### 2.2 Principle of pulse-current waveforms

In this section, the expressions for the established circuit module are determined according to the principles of electric circuits. The generated pulse-current waveform is expressed as a three-segment function based on the established electric circuit model shown in Figure 2. The first segment represents the reverse charging process with an initial current *I*_01_ of 0 A, where four series of PWM sequences operate the switches of the four IGBTs to cause the coil current to increase in the negative direction for obtaining the requested current waveform.

First, IGBT2 and IGBT3 are turned on, whereas IGBT1 and IGBT4 are turned off; the electric current in the TMS coil increases and is determined by the following expression:


(1) $$i_{1}=-\frac{V_{0}}{R}\left(1-e^{-\frac{R}{L}t}\right)+I_{01}e^{-\frac{R}{L}t}$$

where the initial current *I*_01_ = 0 during the initial activation of the IGBTs. This course can be illustrated by the red streamline a in Figure 2. And then IGBT2 is turned off; the electric current of the TMS coil slowly decreases and is given by


(2) $$i_{1}=I_{01}e^{-\frac{R}{L}t}$$

where the initial current *I*_01_ represents the maximum current value at the end of the TMS coil charging process. For this discharging, the current in the TMS coil is shown by the yellow line arrow b in Figure 2. In the following steps, IGBT2 is repeatedly turned on and off, the reverse charging is performed repeatedly. That is, the reverse charging is performed first according to Equation 1 with *I*_01_ ≠ 0 when IGBT2 is turned on, and then done according to Equation 2 when IGBT2 is turned off. The circuit system repeats this process until the peak current of the reverse charging is reached, and the first segment ends at *t* = *t*_1_. During the repeated reverse charging course, a series of PWM sequences, controlled by the editable PWM module, operate the switches of IGBT2 and other IGBTs. These novel technologies are different from those presented by Sorkhabi et al. (2021b) and other scholars (Gattinger et al., 2012; Peterchev et al., 2014; Sorkhabi et al., 2021a, 2022).

For the second segment, IGBT2 and IGBT3 are turned off, and IGBT1 and IGBT4 are turned on. The second segment involves applying a forward voltage to the TMS coil at an initial current *I*_02_, causing the coil to discharge first and then charge forward until the positive peak current is reached at *t* = *t*_2_. Similarly, the second segment is controlled by programmable PWM pulses. The current changes from its maximum negative value to its maximum positive value. During this process, an electric field is induced in the human brain, and depolarization of the cell membrane potential occurs. The electric current in this process can be expressed as


(3) $$i_{2}=\frac{V_{0}}{R}\left(1-e^{-\frac{R}{L}\left(t-t_{1}\right)}\right)+I_{02}e^{-\frac{R}{L}\left(t-t_{1}\right)}$$

where *t*_1_ represents the end of the first segment and *I*_02_ represents the electric current of the TMS coil at *t* = *t*_1_. This current is illustrated by the light-green streamline c in Figure 2.

In the third segment, a reverse voltage is applied to the TMS coil at an initial current *I*_03_, causing a rapid discharge. During the third process, the electric current decreases rapidly to zero via the repeated charging and discharging of the TMS coil. The electric current in the TMS coil can be expressed as


(4) $$i_{3}=-\frac{V_{0}}{R}\left(1-e^{-\frac{R}{L}\left(t-t_{1}-t_{2}\right)}\right)+I_{03}e^{-\frac{R}{L}\left(t-t_{1}-t_{2}\right)}$$

where *t*_2_ represents the time spent on the second segment and *I*_03_ represents the electric current of the TMS coil at *t* = *t*_1_ + *t*_2_. In the third segment, IGBT1 is turned on or off, and the three other IGBTs are turned off. First, the current in the coil decreases in terms of Equation 2 or the second term of Equation 4 when IGBT1 is turned on, resulting in a slowly decreasing current shown by streamline d in Figure 2. And then IGBT1 is turned off to achieve a rapid decreasing current, as shown by the blue streamline e in Figure 2 (Gattinger et al., 2012). And thus, when other IGBTs are turned off, IGBT1 is turned on or off until the current in the TMS coil becomes zero at *t* = *t*_3_.

The pulse-current waveforms shown in Figures 3, 4 can be calculated or simulated using Equations 1–4, and the established circuit model illustrated in Figures 1, 2.

For the simulations of Figures 3, 4, the negative or positive peak current is determined by the required depolarization of the cell membrane potential. Based on the peak current and time of each segment (*t*_1_, *t*_2_, *t*_3_), the slope of each segment is defined. Finally, the duty ratio of PWM and its pulse sequence are modified in terms of the slope of each segment. The optimized quantities are the times (*t*_1_, *t*_2_, *t*_3_) or slopes of the segments.

**Figure 5:**
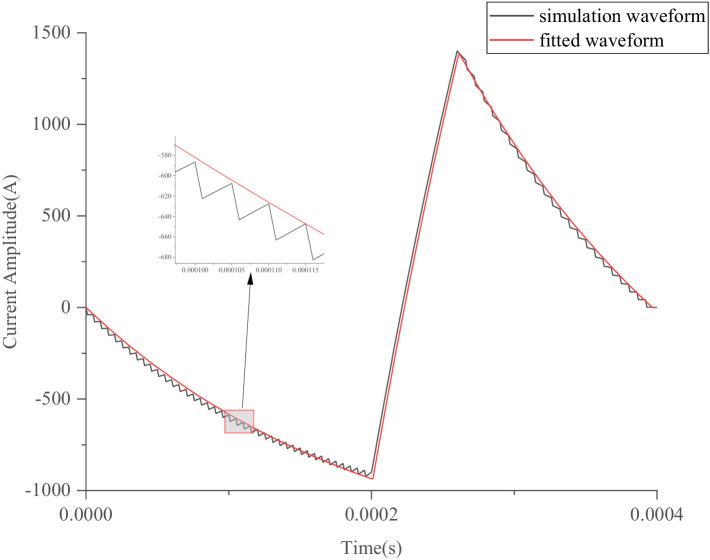
Approximated/fitted waveforms generated by low-frequency IGBT switches.

For a typical charge–discharge process, the slope of the second segment of the pulse waveform remains unchanged because of the time constant *t*_2_ and the required depolarization potential of the neural membrane (negative and positive peaks). However, the slopes of the first and third segments of the pulse-current waveform are defined by the duty cycle of the PWM voltage, which can be adjusted by controlling the on and off states of the four IGBTs in the pTMS system. Thus, modifying the duty cycle of each PWM voltage alters the slopes of the first and third segments. The pulse-current waveform generated by the pTMS system can be approximated using the following three segments:


(5) $$i_{1}=-\frac{k_{1}V_{0}}{R}\left(1-e^{-\frac{R}{L}t}\right),\;0<t<t_{1}$$

(6) $$i_{2}=\frac{V_{0}}{R}\left(1-e^{-\frac{R}{L}\left(t-t_{1}\right)}\right)+I_{02}e^{-\frac{R}{L}\left(t-t_{1}\right)},\;t_{1}<t<t_{1}+t_{2}$$

(7) $$\begin{array}{l} i_{3}=-\frac{k_{3}V_{0}}{R}\left(1-e^{-\frac{R}{L}\left(t-t_{1}-t_{2}\right)}\right)+I_{03}e^{-\frac{R}{L}\left(t-t_{1}-t_{2}\right)},\;\\ t_{1}+t_{2}<t<t_{1}+t_{2}+t_{3} \end{array}$$

where *t*_3_ represents the time spent in the third segment; *k*_1_ and *k*_3_ are the fitted parameters related to the PWM pulse duty ratios (PDR) *t*_1_, *t*_2_, and *t*_3_; and the pulse period *T* = *t*_1_ + *t*_2_ + *t*_3_. Based on Equations 5–7, a circuit model with a low-frequency PWM generator can be established, in which the IGBTs (for example, IGBT1 and IGBT3) turn on and off only once during the first or third segment of the pulse period *T*. Based on these three equations (Equations 5–7), the pulse-current waveform can be obtained, as illustrated in Figure 5 by the red curves.

It is known that a reverse circuit is established, and the TMS coil is charged in reverse when IGBT2 and IGBT3 are turned on (*t* ≤ *t_on_*) (Sorkhabi et al., 2021b; Nilsson and Riedel, 2010), and IGBT1 and IGBT4 are turned off, as shown in Figure 2. The current equation *i*(*t*) can be expressed as


(8) $$i\left(t\right)=-\frac{V_{0}}{R}\left(1-e^{-\frac{R}{L}t}\right),\;t\leq t_{\mathrm{on}}$$

When IGBT2 is turned off (*t_on_* < *t* ≤ *t_on_* + *t_off_*), a discharged circuit is established, and the current equation can be expressed as


(9) $$i\left(t\right)=I_{001}e^{-\frac{R}{L}t},t_{\mathrm{on}}<t\leq t_{\mathrm{on}}+t_{\mathrm{off}}$$

where


(10) $$I_{001}=-\frac{V_{0}}{R}\left(1-e^{-\frac{R}{L}t_{\mathrm{on}}}\right)$$

denotes an original constant.

At *t* = *t_on_* + *t_off_*, the current can be expressed as


(11) $$I_{002}=I_{001}e^{-\frac{R}{L}t_{\mathrm{off}}}$$

The above Equations 8–11 represent a single cycle charging and discharging process of the established circuit. Thus, at *t* = 2 (*t_on_* + *t_off_*), the current is defined by


(12) $$\begin{array}{c} I_{004}=I_{003}e^{-\frac{R}{L}t_{\mathrm{off}}}\\ =\left[-\frac{V_{0}}{R}\left(1-e^{-\frac{R}{L}t_{\mathrm{on}}}\right)-\frac{V_{0}}{R}\left(1-e^{-\frac{R}{L}t_{\mathrm{on}}}\right)e^{-\frac{R}{L}t_{\mathrm{off}}}e^{-\frac{R}{L}t_{\mathrm{on}}}\right]e^{-\frac{R}{L}t_{\mathrm{off}}} \end{array}$$

From Equation 5, we obtain


(13) $$-\frac{k_{1}V_{0}}{R}\left(1-e^{-\frac{R}{L}2\left(t_{\mathrm{on}}+t_{\mathrm{off}}\right)}\right)=I_{004}$$

Therefore, in terms of Equation 13, the fitted parameter *k*_1_ is


(14) $$k_{1}=\frac{e^{-\frac{R}{L}\frac{1-q}{q}t_{\mathrm{on}}}-e^{-\frac{R}{L}\frac{t_{\mathrm{on}}}{q}}}{1-e^{-\frac{R}{L}\frac{t_{\mathrm{on}}}{q}}}$$

where


(15) $$q=\frac{t_{\mathrm{on}}}{t_{\mathrm{on}}+t_{\mathrm{off}}}$$

Similarly, *k*_3_ can be derived from the simulated pulse-current waveform. The simulated waveform defined by Equations 1–4 or Equations 8–12 and the fitted waveform defined by Equations 5–7 are shown in Figure 5. The differences between the simulated and fitted waveforms can be calculated by Equation 16, as follows:


(16) $$\mathrm{RMSE}=\sqrt{\frac{\overset{n}{\underset{k=1}{\sum }}{\left(y_{\mathrm{true}}-y_{\mathrm{pred}}\right)}^{2}}{n}}$$

where *y_true_* denotes the true value of the simulated waveform at the sampling point, and *y_pred_* is obtained from the fitted waveform defined by Equations 5–7.

In Figure 5, *t*_1_ = 200 μs, *t*_2_ = 60 μs, *t*_3_ = 140 μs, *V*_0_ = 400 V, *R* = 0.05 *Ω*, *L* = 10 *μH*, *k*_1_ = 0.185, *k*_3_ = 0.3, and *RMSE* = 33.16 A. The black trapezoidal line illustrates the simulation waveform expressed by Equations 1–4 or simulated by the pTMS high-frequency switching system, which operates according to Equations 1–4. On the other hand, the red line shows the fitted waveform according to Equations 5–7, making it convenient to study the ohmic loss and noise produced by the TMS coil. Although the circuit model established using Equations 5–7 is simple (Nilsson and Riedel, 2010), the voltage *V*_0_ needs to change from *k*_1_*V*_0_ to *V*_0_ and from *V*_0_ to *k*_3_*V*_0_. However, it is inconvenient to change the source voltage *V*_0_.

### 2.3 Electromagnetic modeling and mechanical simulation to reduce noise and ohmic loss

In this section, the TMS coil noise (clicking) and its ohmic loss were considered and calculated.

Two indicators, the ohmic loss *Q* and coil electromagnetic force impulse *P*, were used to describe the performance of the excited pulse-current waveform in reducing the TMS coil noise and its ohmic loss.

According to the principle of the TMS coil ohmic loss *Q*, the generated *Q* of the TMS coil with resistance *R* owing to a pulse current *i*(*t*) (in one period *T*) can be expressed as


(17) $$Q=\overset{T}{\underset{0}{\int }}i^{2}\left(t\right)Rdt$$

Additionally, the magnetic force experienced by current-carrying coil 1 is exerted by TMS coil 2, in terms of Equation 18 as follows:


(18) $$\vec{F}=\frac{\mu_{0}}{4\pi }\int_{c1}I_{1}d{\vec{l}}_{1}\times \int_{c2}\frac{I_{2}d{\vec{l}}_{2}\times {\vec{R}}_{21}}{R_{21}^{3}}$$

where ${\vec{R}}_{21}$ is the distance vector from current element $I_{2}d\vec{l}$ to current element $I_{1}d{\vec{l}}_{1}$ , which is proportional to the square of the coil current *I* = *I*_1_ = *I*_2_ = *i*(*t*), as shown in Equation 19 owing to the series coils:


(19) $$\vec{F}\propto I^{2}$$

Because the magnetic force impulse $\vec{P}$ experienced by the TMS coil is an integral of $\vec{F}$ over one time period *T*, the value of $\vec{P}$ is also proportional to *i*^2^(*t*):


(20) $$\vec{P}=\overset{T}{\underset{0}{\int }}\vec{F}dt$$

As shown in the aforementioned Equations 17–20, *Q* and $\vec{P}$ are integrals of *i*^2^(*t*) over the duration of the TMS coil pulse current, and thus, *Q* and $\vec{P}$ can reach their minimum values simultaneously.

**Figure 6:**
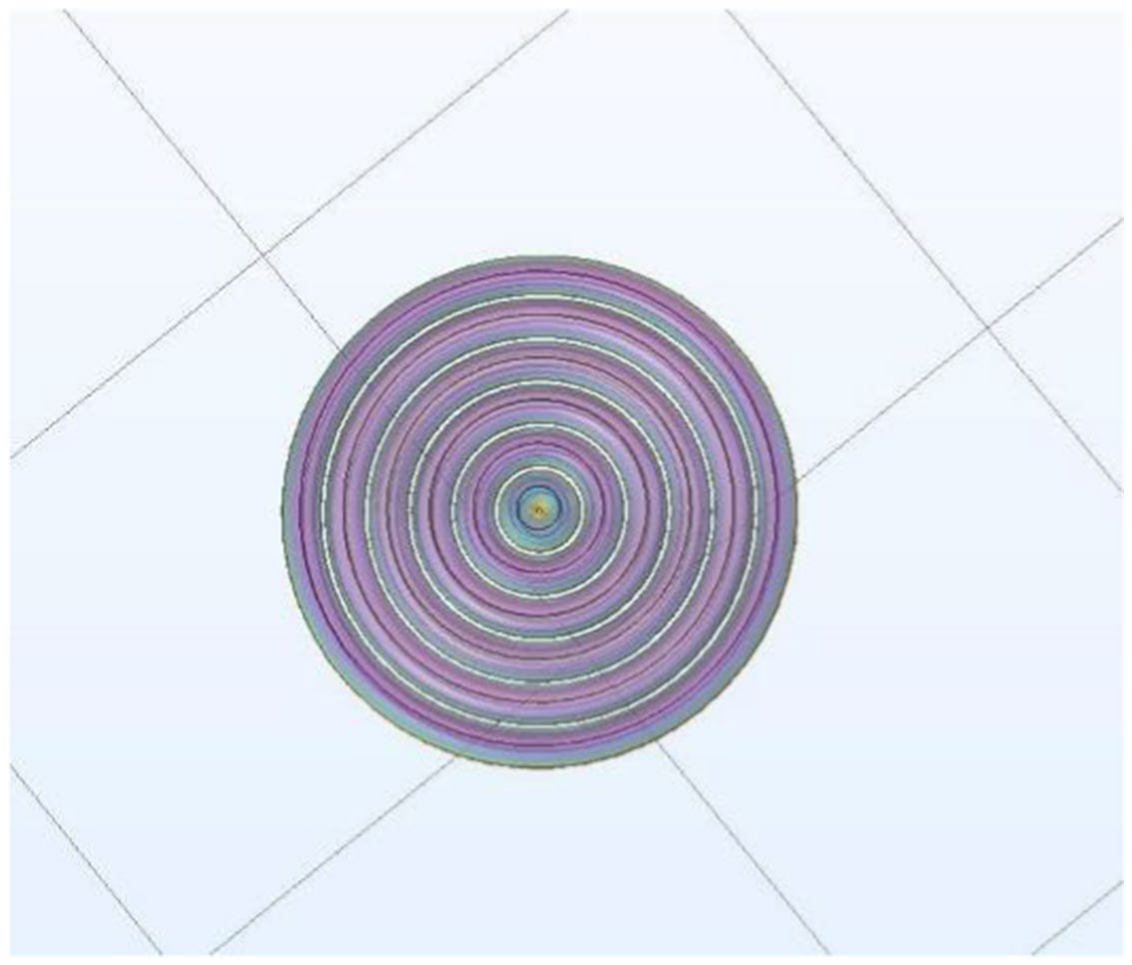
Designed TMS coil model in COMSOL.

The values of *Q* and $\vec{P}$ can be calculated using Equations 8, 20 after the pulse current *i*(*t*) is simulated using MATLAB Simulink based on the established circuit model. The values of *Q* and $\vec{P}$ can also be calculated using the COMSOL software by inputting the simulated pulse current *i*(*t*). In this study, the applied excitation was a pulsed electric current generated using the pTMS circuit model mentioned earlier. First, a concentric TMS coil with radii of 4–44 mm, step length of 8 mm, and six turns was designed to simulate the impulse $\vec{P}$ and force $\vec{F}$ experienced by a conventional concentric coil. Subsequently, the magnetic force $\vec{F}$ experienced by the TMS coil was simulated using the COMSOL software. The designed TMS coil model is illustrated in Figure 6. Finally, the impulse $\vec{P}$ of the 6-coil model in the pulse period *T* was calculated to characterize the vibration energy or the noise energy (clicking) of the coils.

### 2.4 Sensitivities of pulse-current waveform

In this section, a series of pulse-current waveforms were simulated by adjusting the pTMS system parameters, and the magnetic force and its impulse were calculated.

#### 2.4.1 Effect of source voltage *V*_0_

**Figure 7:**
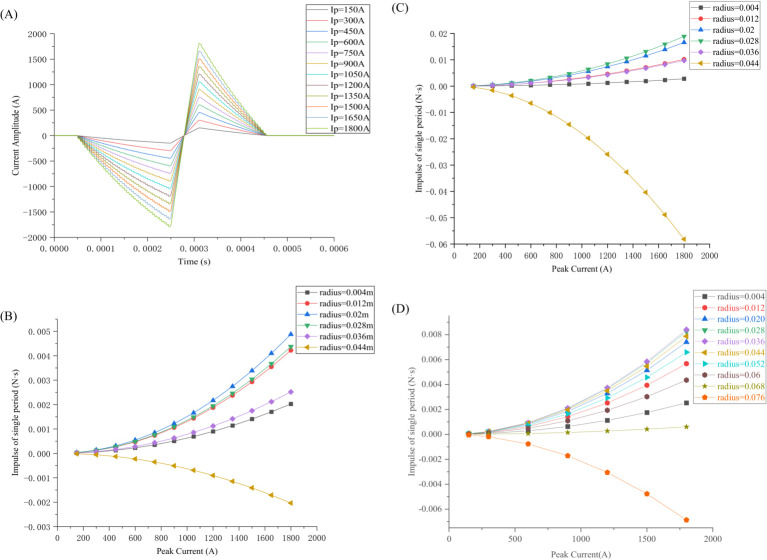
Pulse-current waveforms and magnetic force impulse varying with peak current: **(A)** Pulse-current waveforms varying with source voltage; **(B)** Magnetic force impulse varying with *I_p_* for various radii simulated in COMSOL (6 turns); **(C)** Magnetic force impulse varying with *I_p_* for various radii calculated by Equation 20 (theoretical calculations); **(D)** Magnetic force impulse varying with *I_p_* for various radii calculated in COMSOL (10 turns).

Based on the established circuit shown in Figure 2, the calculated pulse-current waveforms are illustrated in Figure 7A. These waveforms vary with the DC source voltage *V*_0_ when the PDR, *t*_1_, *t*_2_, *t*_3_, *R,* and *L* are given. As shown in Figure 7A, the peak value *I_p_* of the pulse-current waveform increases with increasing source voltage *V*_0_, and *I_p_* is defined primarily by the source voltage *V*_0_.

The magnetic force impulse is illustrated in Figure 7B. Because the magnetic force *F*(*t*) is proportional to *i*^2^(*t*), the magnetic force impulse *P* experienced by the TMS coil increases linearly with *i*^2^(*t*). Simultaneously, increasing the excitation current results in increased heat loss *Q*, following the square law. Therefore, decreasing the source voltage *V*_0_ or excitation current *I_p_* effectively reduces the ohmic and vibration energy losses of the coil. However, decreasing the source voltage *V*_0_ causes the pulse peak value *I_p_* to decrease, resulting in the induced electric field intensity in the human brain not being sufficiently strong to depolarize the neural membrane. The source voltage *V*_0_ should not be significantly low and must satisfy the depolarization potential of the neural membrane.

As shown in Figure 7B, the impulse $\vec{P}$ increases as the peak current *I_p_* increases, with the impulse $\vec{P}$ experienced by the TMS coil at a radius of 0.02 m being the maximum, while the impulse $\vec{P}$ experienced by the outermost circle is in the opposite direction.

In Figure 7C, the electromagnetic force impulse exerted upon each coil of the six-turn coil is calculated in terms of Equation 20, i.e., theoretical calculations. The calculation results are in good agreement with those simulated by COMSOL, as shown in Figures 7B,C: (1) The calculated impulses increase with the current increasing from 200 A to 1,800 A. Similarly, the simulated impulses also increase with the current increasing from 200 A to 1,800 A. (2) As the radius of the coil increases, the impulse or force exerted upon each coil first increases and then decreases until it reaches zero, and finally increases in the opposite direction. (3) The calculation results are slightly larger than the simulated values. A possible reason for this discrepancy is that in the calculation, the current was concentrated on a point or a line with its dimension (radius) = 0.

Similarly, we changed the number of the TMS coils from 6 turns to 10 turns and obtained the same conclusion, as demonstrated in Figure 7D. The direction of the force or impulse exerted upon the outermost coil is opposite to the direction of the force or impulse exerted upon the innermost coil. From Figures 7B–D, it is inferred that the force or impulse exerted upon the TMS coil first increases, then decreases until it reaches zero, and finally increases in the opposite direction.

#### 2.4.2 Effect of TMS coil inductance *L*

**Figure 8:**
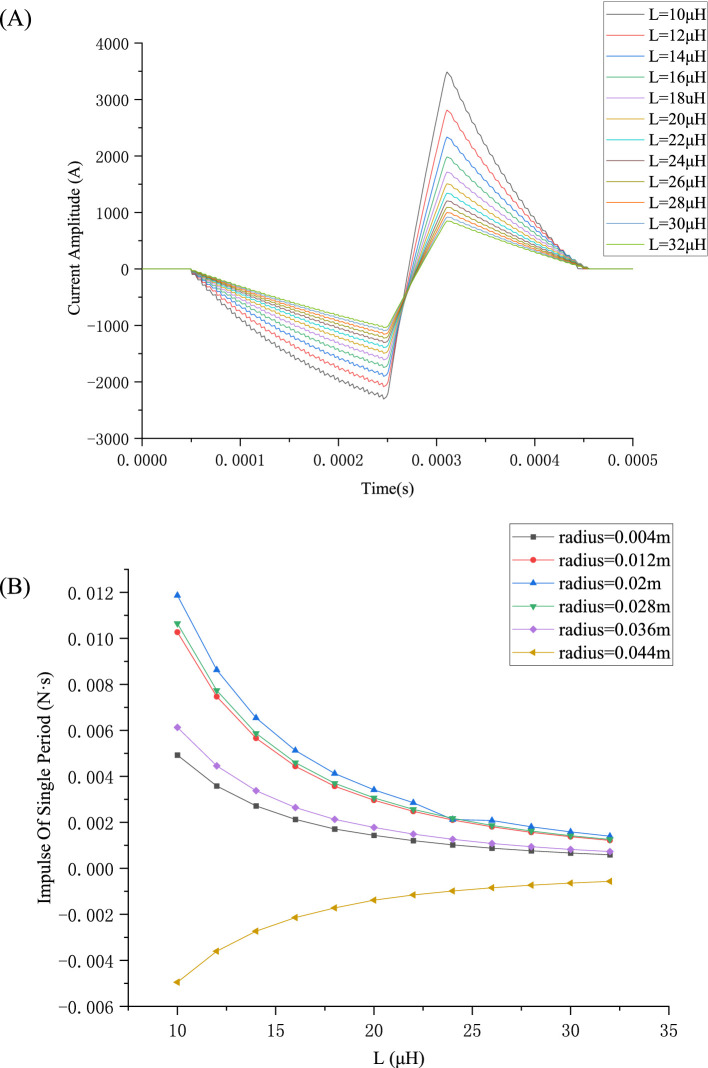
Pulse-current waveforms and magnetic force impulse variation with *L*: **(A)** Pulse waveforms with varying *L*; **(B)** Magnetic force impulse varying with *L* for various radii.

When the source voltage *V*_0_ is given, the peak value *I_p_* of the pulse current *i*(*t*) varies with inductance *L* of the TMS coils, as shown in Figure 8A. It can be observed that the peak value *I_p_* of the pulse current *i*(*t*) increases as the inductance *L* of the TMS coils decreases, with the excited pulse currents in the TMS coil varying as the coil inductance *L* varies from 10 μH to 32 μH. The corresponding magnetic force impulse $\vec{P}$ is illustrated in Figure 8B. The relationship between the magnetic force *F* experienced by the TMS coil and its inductance *L* follows a power function, where the magnetic force *F* or impulse $\vec{P}$ decreases as the TMS coil *L* increases. Likewise, the excited current amplitude (peak value) *I_p_* decreases as the TMS coil *L* increases. Therefore, to ensure that the current is sufficient to fulfil the stimulation requirements without generating excessive magnetic force impulse $\vec{P}$ (which can cause significant noise), the TMS coil inductance *L* can be considered for a compromise. In this study, *L* within the typical range of 20–25 μH was considered.

From Figure 8B, it is evident that the peak *I_p_* decreases as the TMS coil *L* increases, leading to a decrease in the impulse $\vec{P}$ .

In this study, the impedance included mainly the TMS coil resistance and its inductance (the power and its capacitance are not included in this investigation). The effect of the coil inductance *L* on the pulse waveform varies with *L*. The inductance of the coil has an impact on the current waveform, affecting not only the current peak *I_p_* but also the shape (slope) of the current waveform. The current peak *I_p_* decreases with increasing *L*, and the slope of the current waveform also follows this pattern, as shown in Figure 8A. On the other hand, the exerted coil force or impulse decreases with increasing *L*. The effect of the TMS coil resistance on the energy loss is almost linear. Moreover, the resistance is usually so small that this study will not focus on its effects.

#### 2.4.3 Effects of single- and dual-phase currents

**Figure 9:**
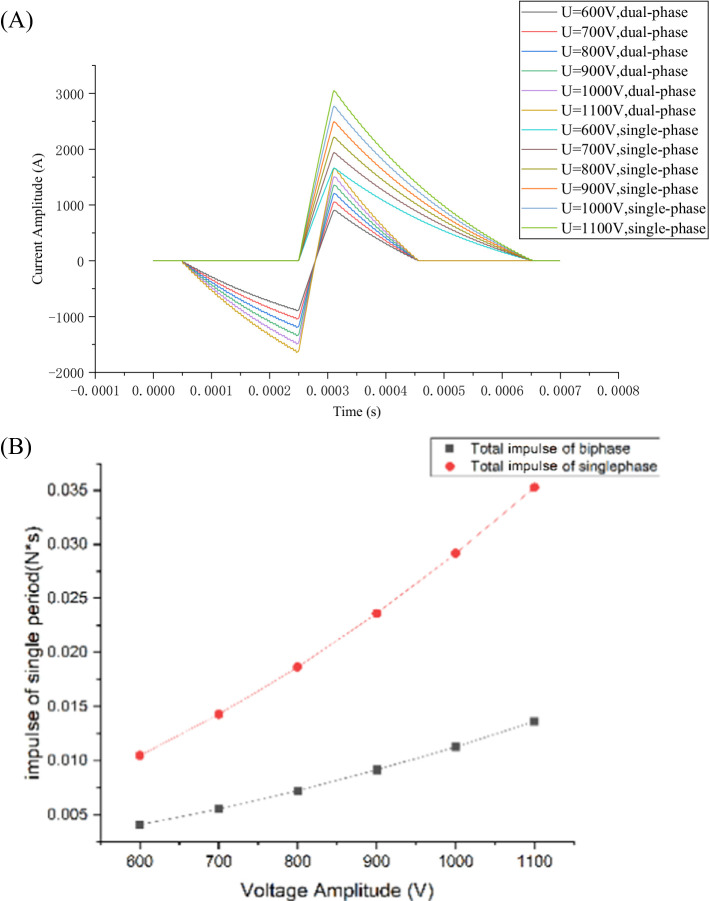
Monophasic and biphasic waveforms and experienced impulses: **(A)** Monophasic and biphasic pulse waveforms; **(B)** Magnetic force impulse for monophasic and biphasic pulse waveforms.

Figure 9A shows a group of single-phase and dual-phase excitation currents with the same pulse width and peak-to-peak current (IPP). The magnetic force impulse $\vec{P}$ was calculated, as shown in Figure 9B. Regardless of the number of phases of the excited current, whether single-or dual-phase, the impulse $\vec{P}$ experienced by the TMS coil increased with the source voltage *V*_0_. However, the impulse $\vec{P}$ generated by the single-phase current waveform was 2.58 times greater than that produced by the biphasic waveform. This indicates that the biphasic current waveform has a significant advantage in reducing TMS noise (clicking) owing to the lower experienced impulse *P*.

As shown in Figure 9B, the impulse $\vec{P}$ generated by the biphasic current waveform is less than that generated by the single-phase current waveform under a given source voltage *V*_0_. The difference in impulse $\vec{P}$ between the two types of waveforms became more significant as the voltage increased. Compared with the single-phase current waveform, the biphasic waveform had a negative bias, which reduced the peak value *I_p_* of the second segment of the pulse-current waveform and the electromagnetic impulse $\vec{P}$ experienced by the TMS coil system, effectively reducing noise.

#### 2.4.4 Effect of symmetric pulse currents

**Figure 10:**
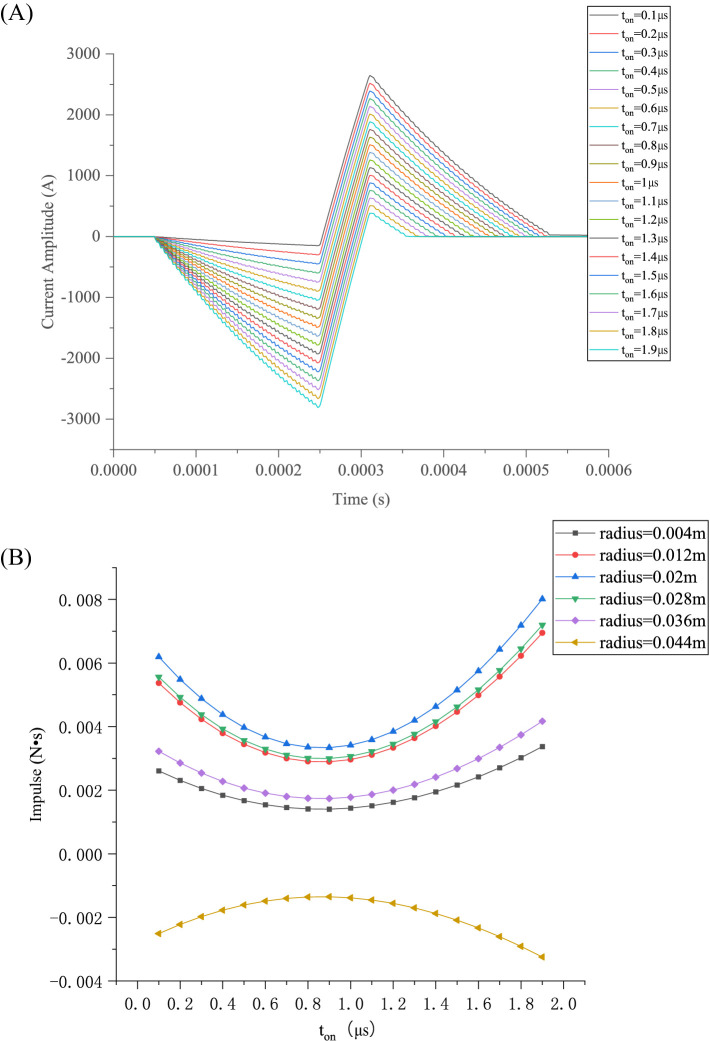
Pulse-current waveforms and magnetic-force impulse varying with duration of narrow pulse: **(A)** Pulse waveform variation with *t*_on_; **(B)** Magnetic force impulse varying with *t*_on_ for various radii.

Figure 10A illustrates a group of asymmetric current waveforms varying with *t*_on_ under the same IPP, where *t*_on_ denotes the time the IGBT is on and can specify the degree of asymmetry of the pulse-current waveform. When *t*_on_ = 1 μs, a symmetric pulse-current waveform is excited, where the positive peak value of the pulse current equals the negative peak value. A set of asymmetric current waveforms was simulated to study the effect of waveform symmetry on the magnetic force impulse, and the corresponding magnetic force impulse $\vec{P}$ was calculated, as shown in Figure 10.

In the circuit model, *t*_on_ has a specific meaning; *t*_on_ represents the duration of the narrow pulse, and its unit is μs. When *t*_on_ = 1 μs, the waveform is symmetric, as shown in Figure 10.

According to Equation 14, *k*_1_ increases with *t_on_*, causing the negative peak of the current waveform to become increasingly biased in the negative direction. When *t_on_* increases, the magnitude of $\vec{P}$ experienced by the TMS coils at various radii in six turns initially decreases and then increases, reaching its minimum value nearly at *t_on_* = 0.9 μs, with the minimum heat *Q* and vibration energy.

**Figure 11:**
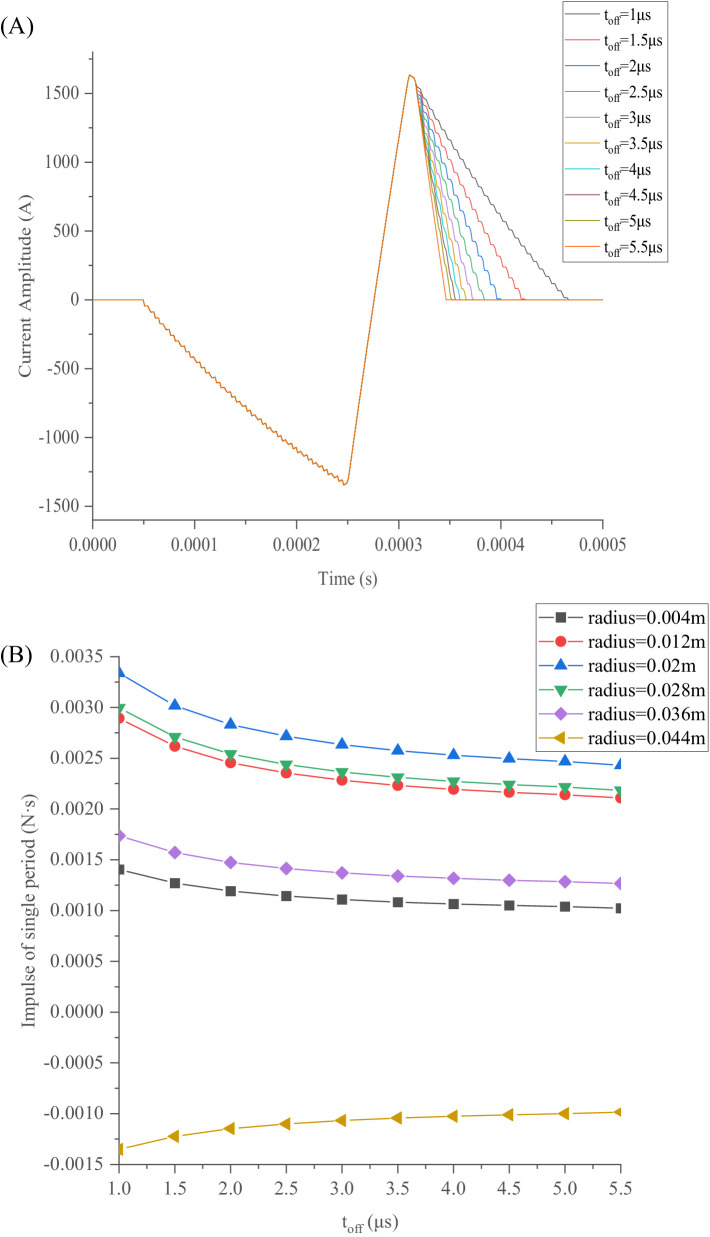
Pulse-current waveforms and magnetic force impulse varying with *t*_off_: **(A)** Third segment of pulse waveform varying with *t*_off_; **(B)** Magnetic force impulse varying with *t*_off_ for various radii.

As demonstrated in Figure 10B, the magnetic force impulse experienced by the TMS coils is smallest when *t*_on_ = 0.9 μs. Therefore, the effect of *t*_off_ on the slope of the third segment under *t*_on_ = 0.9 μs can be investigated. The slope of the third segment of the current pulse waveform increases with *t_off_*, as shown in Figure 11A, while the first and second segments of the pulse waveform and the IPP remain unchanged. The value of *t*_off_ indicates the time of IGBT shutdown in one cycle. When the IGBT is shut down, the circuit resistance becomes extremely high, such that the current of the TMS coil decays rapidly, according to Equation 2, resulting in a larger slope in the third segment of the current pulse waveform. A larger slope reduces the impulse experienced by each turn of the coil, as shown in Figure 11B.

The intensity of the excited current, the shape of the excited pulse-current waveform, the duration, and its symmetry have effects on the magnetic force or impulse *P* experienced by the TMS coil. Additionally, the ohmic loss *Q* is dependent on the current *i*^2^(*t*) and the resistance *R* of the TMS coil. Notably, *Q* can reach its minimum value for a given TMS coil when the experienced impulse *P* is at its minimum.

### 2.5 Optimization

To minimize the impulse *P* (the magnitude of $\vec{P}$ ) and the ohmic loss *Q*, the shape of the biphasic pulse-current waveform—defined primarily by the PDR of PWM signals (*k*_1_, *k*_3_), and the parameters *t*_1_, *t*_2_, and *t*_3_—was mathematically modeled and optimized using an optimization algorithm because the other parameters such as the TMS coil *R* and *L*, source voltage *V*_0_, and the total pulse time period *T* were predefined when the TMS circuit system was established and remained nearly constant.

**Figure 12:**
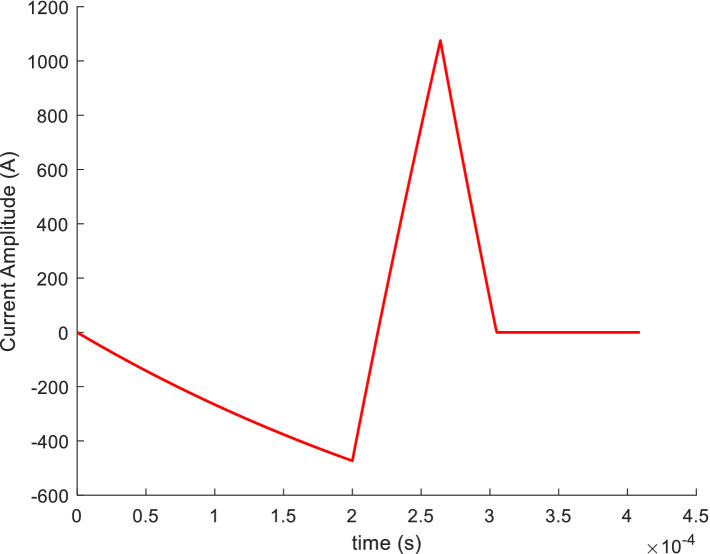
Optimized waveform.

In general, the optimized waveform, calculated using the particle swarm optimization algorithm, is a symmetric pulse-current waveform, such as a sinusoidal or triangular waveform. However, recent studies have demonstrated that asymmetric waveforms may outperform conventional symmetric waveforms. Therefore, in this study, *P* or *Q* was taken as the objective function; variables *k*_1_, *k*_3_, *t*_1_, *t*_2_, and *t*_3_ were optimized; and the circuit structure parameters such as *V*_0_, *R,* and *L* remained constant. To determine *k*_1_, *k*_3_, *t*_1_, *t*_2_, and *t*_3_ at which *P* or *Q* reached the minimum value, the particle swarm optimization algorithm was applied. The values of *k*_1_ and *k*_3_ range from zero to one, as defined by the PDR of the PWM voltage. The optimized waveform was obtained when the experienced impulse *P* reached the minimum, as shown in Figure 12, where *k*_1_ = 0.1203, *k*_3_ = 1, *t*_1_ = 200 μs, *t*_2_ = 64 μs, *t*_3_ = 40 μs. The rapid descent of the third segment allows the impulse *P* to reach its minimum under an unchanging IPP. This ensures that the induced electric field can effectively drive ion currents on the nerve membrane, resulting in local hyperpolarization or depolarization of the neuronal excitable membrane (Aberra et al., 2020) at the minimum impulse noise.

The waveform optimized by the particle swarm algorithm was divided into three segments. The first and third segments generated a negatively induced electric field on the nerve cell membrane, corresponding to the direction in which the induced electric field polarizes the nerve cell membrane. The second segment generated a positively induced electric field on the nerve cell membrane, corresponding to the direction in which the induced electric field depolarizes or hyperpolarizes the nerve cell membrane. Studies have increasingly demonstrated that an induced electric field is effective in TMS when it depolarizes the nerve membrane, hyperpolarizes, and stimulates action potentials after reaching the required threshold. The change in the potential of the nerve cell membrane can be explained using an integral emission model (Koponen and Peterchev, 2020). In this model, the induced electric field initially depolarizes the membrane potential and then hyperpolarizes it. Finally, the nerve cell generates a pulse current owing to the induced electric field acting across the nerve cell membrane when the expected threshold of depolarization is reached. Following this sequence, the nerve cell membrane enters a refractory period during which it no longer responds to the induced electric field (Dou et al., 2012). Therefore, the third segment of the optimized waveform becomes functionally redundant, and its current should be reduced to zero at the fastest speed to minimize ohmic loss and vibration energy.

**Figure 13:**
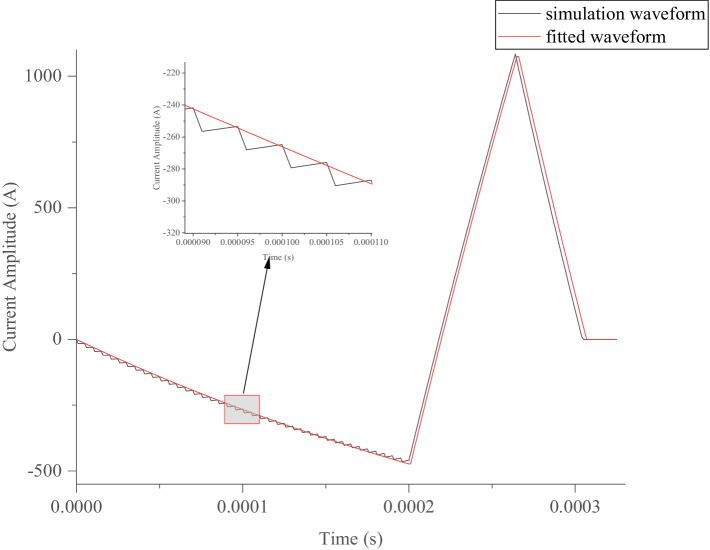
Optimized simulated pulse-current waveform and its fitted pulse waveform with RMSE of 26.59 A.

The optimized waveform generated by the particle swarm algorithm has been implemented in clinical practice. In the pTMS system, by setting the PDR of the first-and third-segment PWM voltages, the optimized waveform was simulated using Equations 1–4 and the established model shown in Figure 2. The simulated results closely matched those calculated from Equations 5–7, as demonstrated in Figure 13, where *t*_on_ = 0.62 μs, *t*_off_ = 5.38 μs, and *q* = 0.10 for the first segment, and *t*_on_ = 0 μs, *t*_off_ = 6 μs, and *q* = 0 for the third segment.

In Figure 13, the red line indicates the excitation waveform obtained by Equations 5–7, and the black line depicts the simulated pulse-current waveform from the pTMS circuit system. The two waveforms closely match, with an *RMSE* of 26.59 A.

#### 2.5.1 Optimization results

**Figure 14:**
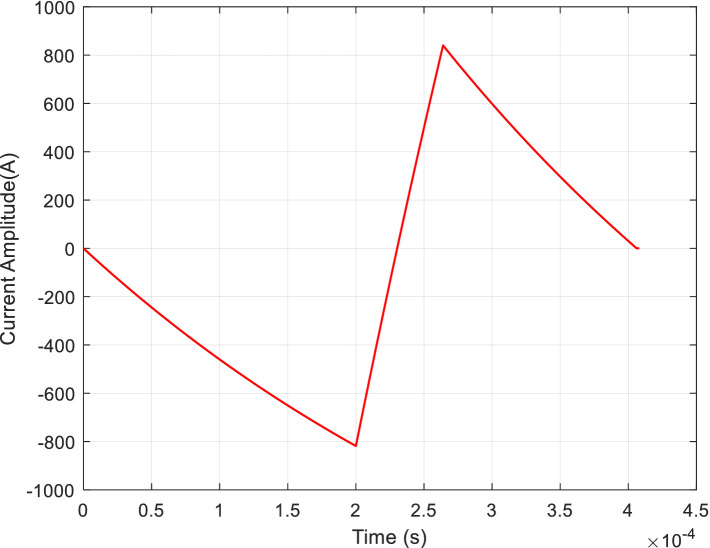
Conventional pulse-current waveform.

Subsequently, the ohmic loss of the TMS coil was calculated and simulated based on Equation 17. The results demonstrated a 40% reduction in ohmic loss for the optimized pulse-current waveforms compared to that observed for the conventional symmetric triangular waveform (from 4.42 J to 2.58 J), as shown in Figure 14. The optimized waveform has more advantages: it has a higher current peak, leading to a significant membrane potential change, which ensures a better stimulation effect. The asymmetric waveform may lead to a lower ohmic loss and a reduced experienced impulse *P* (clicking noise).

**Figure 15:**
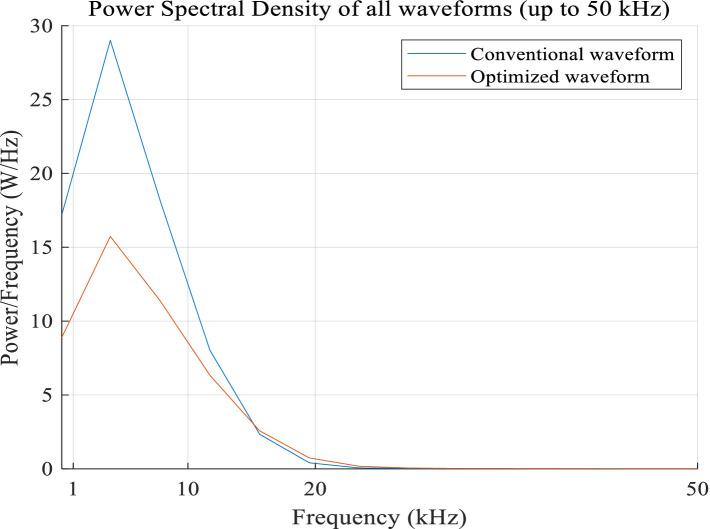
Optimized and conventional square current spectra.

It is well known that the noise generated by the TMS coil is caused by the pulsed magnetic force it experiences, which is proportional to the cross-product of the current of the current-carrying TMS coil and the magnetic field. Therefore, the pulsed magnetic force is proportional to the square of the coil current, indicating that the noise (clicking) is related to the square of the current in the TMS coil. Thus, the pulse noise generated by a TMS coil can be studied using the spectrum of the square of the current in a current-carrying TMS coil. The power spectra of the optimized pulse current, illustrated in Figure 13, and the conventional symmetric triangular pulse waveform, shown in Figure 14, can be calculated as shown in Figure 15.

It can be observed that at most frequencies, especially within a larger range near the peak frequency, the optimized current waveform energy is much smaller than that of the traditional current waveform in Figure 15. Herein, the optimized waveform peak is obtained at 3.9 kHz with a peak of 15.7 W/Hz, whereas the conventional waveform peak is obtained at 3.9 kHz with a peak of 29 W/Hz. Thus, the energy in one cycle decreases from 88.48 J (the conventional waveform) to 51.61 J (the optimized waveform). Additionally, the energy distribution tends toward high frequencies, which is the direction toward which our future research endeavors are headed: to make the peak frequency spectrum of the square current exceed the range of human auditory perception via the use of a certain technology. It can be seen that the optimized waveform has lower energy loss, including *P* and *Q* in the frequency domain.

## 3 Results and discussion

This study presents a novel and applicable circuit model that can generate various pulse-current waveforms. The research focused on an asymmetric pulse-current waveform optimized using the particle swarm optimization algorithm. This optimized waveform results in a smaller ohmic loss and reduced vibration noise (clicking) in TMS coils compared to those experienced with conventional waveforms, such as the symmetric triangular waveform. The circuit model presented herein consists of several key components, which were developed as follows. First, a rectifying unit was established to convert the AC into DC and store the electrical field energy in the capacitors. Second, in accordance with expected requirements, a programmable control unit was designed to generate a series of control codes to create a type of PWM. Finally, the PWM, which is a series of programmable codes, was output to control the on and off states of the four IGBTs to obtain the required pulse-current waveform.

The pulse-current waveform generation method used in this study differs from traditional methods. Traditional methods often rely on capacitor resonance and adjusting impedance parameters to generate optimized waveforms. By contrast, this study employed a non-resonant, high-frequency switch circuit to generate the expected pulse-current waveforms, which is a new method of flexibly adjusting the slope of the pulse-current waveform using high-frequency PWM voltage pulses to control the IGBTs.

The pulse-current waveform was calculated and simulated, and the effects of several key parameters were studied. First, the effects of several circuit parameters, such as the source voltage *V*_0_ and TMS coil inductance *L*, on the pulse-current waveform were simulated and studied. The peak value of the pulse current increased with *V*_0_ and decreased with *L* because *R* is exceedingly small that it can often be neglected. Second, the geometric characteristics (*k*_1_, *k*_3_, *t*_1_, *t*_2_, *t*_3_) of the pulse-current waveform and their effects on the heating and noise (vibration, clicking) of the TMS system were investigated. Proper biasing and symmetry adjustments of the pulse-current waveform greatly improved its performance compared to that of the symmetrical waveform in reducing coil heating and vibration energy, and provided advantages in terms of increasing the neural membrane potential variation. To minimize ohmic loss *Q* and impulse *P*, this study used the particle swarm optimization algorithm, which was validated via theoretical analysis and simulations. Finally, the optimized pulse-current waveform and its parameters (*k*_1_, *k*_3_, *t*_1_, *t*_2_, *t*_3_) were obtained as follows: *k*_1_ = 0.1203, *k*_3_ = 1, *t*_1_ = 200 μs, *t*_2_ = 64 μs, *t*_3_ = 40 μs.

The optimized pulse-current waveform was inferred to have three stages. In the first stage, the current decreases slowly, creating a weak induced electric field that further polarizes the nerve membrane. This gradual current reduction prevents excessive current on the coil during the rising phase. In the second stage, the rapidly rising current generates a strong induced electric field that depolarizes the nerve membrane and hyperpolarizes it to a threshold, triggering an action potential. Finally, in the third stage, the current rapidly drops, restoring the TMS system to a zero state, as the nerve cells enter a refractory period during which the induced electric field generated by the time-varying magnetic field has no effect.

Waveforms optimized using the particle swarm algorithm can be implemented in clinical practice. The pTMS system and circuit model were established by setting the PDR of the first-and third-segment PWM voltages, and the parameters that may specify the shape of the pulse-current waveform, especially the slope of the first and third segments, were found to be *t*_on_ = 0.62 μs, *t*_off_ = 5.38 μs, and *q* = 0.10 for the first segment, and *t*_on_ = 0 μs, *t*_off_ = 6 μs, and *q* = 0 for the first, third, and third segments, respectively. Additionally, a spectrum analysis of the optimized waveform was conducted and revealed the spectrum peak to be 15.7 W/Hz at a frequency *f* = 3.9 kHz for the optimized waveform, which is 29 W/Hz lower than that of the conventional symmetric triangular waveform, and thus, the total energy loss in one cycle decreases from 88.48 J (for the conventional waveform) to 51.61 J (for the optimized waveform). However, this result may not be the optimal one, and there may be other waveforms that can achieve even lower energy losses, which is the direction for our future research endeavors: how to adjust the width of PWM pulses to flexibly change the slopes of the three segments, especially to quickly decrease the current in the coil in the third segment. In addition, the frequency (or narrow pulse) used in this study was very high (short, < 10 μs), close to the highest frequency of IGBT switches, which may easily damage the device. In the future, we will strive to reduce the switching frequency and conduct experimental research on obtaining the minimum noise and heat loss.

## Data Availability

The original contributions presented in the study are included in the article/supplementary material, further inquiries can be directed to the corresponding authors.
